# Tuberculosis

**Published:** 1890-02

**Authors:** 


### TUBERCULOSIS.

The reports of the *Fifth International Veterinary Congress*, held in Paris, credited Prof. Liautard with the statement that "the tuberculosis makes fearful ravages in the United States of "America, and that it might be affirmed that 25 per cent. to 50 "per cent. (of cattle) were affected, and they were often animals "of very great value." And again : "Every year there died of "phthisis from 500,000 to 600,000 people." We did not need to be assured, as we have been, by a personal communication from Prof. Liautard that he did *not* make such a statement. In speaking of the extent to which the disease existed in certain sections of the country, what he did say, was, "that in certain herds, in dairies "close to our large cities, 25 per cent. to 50. per cent. of the animals "were affected."

The mis-interpretation of his remarks and their publication in numerous foreign journals, as well as some in our own country, is more than an unfortunate error ; it is a certain damage to the cattle interests of the country.

We take pleasure in publishing the following letter of Dr. Williamson Bryden, of Boston, live stock inspector for British Steamships, who examines the greater majority of the cattle which are shipped from this country to Europe.

In refutation of any misconception of Dr. Liautard's statement, we trust that all foreign journals will make note of Dr. Bryden's letter in order to place the importance of its contents before their government and the public interested in cattle commerce.

*To the Editors of the*
JOURNAL OF COMPARATIVE MEDICINE AND VETERINARY ARCHIVES.

DEAR SIRS :—I was very much surprised on receiving the "Veterinary Journal" (London) for December, at Prof. Liautard's remarks *in re.* tuberculosis, page 457. Dr. Liautard remarks that the "disease made fearful ravages in the United States of America. It might be affirmed that 25 to 50 per cent. were affected, &c." For the last ten years or more I have yearly inspected from 25,000 to 50,000 head of our cattle from Boston, Portland and several cargoes from New York, for export per British steamships. This year alone 75,000 head.

My experience has been that with the exception of a few cases each year of actinomycosis among distillery and slop-fed animals, the disease has been entirely absent, or we would have heard of it from the inspectors and butchers of London, Liverpool and Glasgow, as the cargoes consisted of steers, bulls, oxen, cows and heifers—grass-fed, grain-fed and distillery-fed. Dr. Burr, inspector of dead meats for our Board of Health, informs me that for ten weeks, from October 1st, he had found at the Brighton abattoir only about six cases out of about 10,000 cattle inspected after slaughter. Abattoir statistics probably show less than the number diseased; still in my opinion 10 per cent. of those killed outside of city limits and at private slaughter houses would be sufficiently alarming and quite high enough. Now, as the cattle slaughtered at Brighton and the cattle shipped from here are probably a fair average of the cattle of the United States, *excepting the milk cows and calves*, the statement at once shows itself to be preposterous.

In the census of 1880, the United States had 12,500,000 milk cows, and about 24,000,000 other cattle. As the percentage among milk cows is always far greater than among other cattle, the inference would be that about all the cows in the country are tuberculous, especially so where we take a State like Massachusetts with 150,000 milk cows and 110,000 other cattle.

The statement is mischievous and unfair to our stock raisers. It fosters more firmly sanitary restrictions of foreign governments which it was hoped would soon be modified, as they cause us now a loss of about $15 a head. This year 100,000 live cattle have been shipped *from Boston alone.* The loss from this cause is, therefore, about $1,500,000. As this is probably about one-quarter of the shipments of live stock from this country, it becomes a subject of vast importance, especially when we reflect that most of our large ships carry twice as many carcasses in the refrigerators as they do alive, and then add canned and other products that are likewise affected, and the result shows the necessity for prudent and cautious statements, especially from those whom many may suppose are representatives of our profession, whether they are or not.

There is so much being said about tuberculosis at present, that our young men are losing their heads over it, and every barn they enter and hear a cow cough, they pronounce suspicious. If I owned a fine herd I would rather have a *little tubercle or two* among them, than some professional men I have heard of lately. Such a blunder is humiliating to the profession in this country.

Yours, very truly,

*Boston, Dec. 29th, 1889.* WILLIAMSON BRYDEN.

